# The Relationship between Subjective Cognitive Decline and Health Literacy in Healthy Community-Dwelling Older Adults

**DOI:** 10.3390/healthcare8040567

**Published:** 2020-12-16

**Authors:** Akio Goda, Shin Murata, Hideki Nakano, Koji Nonaka, Hiroaki Iwase, Kayoko Shiraiwa, Teppei Abiko, Kunihiko Anami, Jun Horie

**Affiliations:** 1 Department of Physical Therapy, Faculty of Health Sciences, Kyoto Tachibana University, Kyoto 607-8175, Japan; murata-s@tachibana-u.ac.jp (S.M.); nakano-h@tachibana-u.ac.jp (H.N.); shiraiwa@tachibana-u.ac.jp (K.S.); abiko@tachibana-u.ac.jp (T.A.); horie-j@tachibana-u.ac.jp (J.H.); 2 Department of Rehabilitation, Faculty of Health Sciences, Naragakuen University, Nara 631-8524, Japan; nonaka@naragakuen-u.jp (K.N.); anami@naragakuen-u.jp (K.A.); 3 Department of Physical Therapy, Faculty of Rehabilitation, Kobe International University, Kobe 658-0032, Japan; iwase@kobe-kiu.ac.jp

**Keywords:** community-dwelling older adults, health literacy, preclinical Alzheimer’s disease, subjective cognitive decline

## Abstract

Few studies have examined the effects of health literacy on people at risk of developing dementia; its effects on the pathogenesis of subjective cognitive decline (SCD) are particularly unclear. This study aimed to clarify the relationship between health literacy and SCD in a population of healthy community-dwelling older adults. SCD status was assessed using the Cognitive Function domain of the Kihon Checklist (KCL-CF). Health literacy, in turn, was evaluated using the Communicative and Critical Health Literacy (CCHL) scale. Global cognitive function and depressive symptoms were evaluated using the Mini-Mental State Examination (MMSE) and a five-item version of the Geriatric Depression Scale (GDS-5), respectively. Participants who were suspected of having SCD were significantly older than their non-SCD peers, and scored significantly worse on the CCHL, MMSE, and GDS-5. In addition, SCD status was found to be associated with CCHL and GDS-5 scores, as well as age, according to a logistic regression analysis. These findings suggest that low health literacy is linked to SCD morbidity in healthy community-dwelling older adults and should prove useful in the planning of dementia prevention and intervention programs for this population.

## 1. Introduction

The percentage of the older adult population living with dementia is growing in combination with increasing international life expectancies [1]. Dementia is associated with a high risk of disability and, consequently, death in older adults [2]. Therefore, preventing the onset of dementia is an urgent challenge that must be addressed to reduce this disease’s burden on the general population [3]. However, evidence for dementia therapies is scarce, with little progress being made in developing effective drugs for the treatment of this disease [4]. The targeting of potentially modifiable risk factors shared by dementia patients to prevent or delay the disease’s onset is considered the most effective contemporary strategy for reducing their numbers [5]. Traditionally, dementia is analyzed based on a framework divided into the stages of normal health and dementia, which are both bridged by an intermediate stage called mild cognitive impairment (MCI) [6]. Interest has grown in subjective cognitive decline (SCD), a preclinical asymptomatic stage of dementia believed to be a precursor to MCI [7]. Despite having no difficulties with daily living activities, and showing a normal level of performance on objective cognitive tests, SCD patients experience reductions in their subjective cognitive functioning [8] and are reportedly at a greater risk of developing MCI and Alzheimer’s disease when compared to healthy older adults [7,9,10]. These connections imply that SCD patients are an optimal target for early intervention strategies [11].

The relationship between cognitive activity and health literacy has received attention in discussions about the prevention of cognitive decline in older adults [12,13]. Health literacy has been defined by the World Health Organization (WHO) as “cognitive and social skills which determine the motivation and ability of individuals to gain access to, understand, and use information in ways which promote and maintain good health” [14]. People with high health literacy tend to participate in cognitive, social, and physical activities, all of which have demonstrated a good effectiveness in maintaining cognitive functions [15,16,17], with this ability helping to indirectly prevent dementia. However, the amount of research on health literacy among individuals at risk of dementia remains limited [13,18]. Although some reports, to date, have looked at health literacy’s effects on peoples’ susceptibility to MCI [12,19] and dementia [20,21,22], none have thus far examined its connection with SCD risk.

Clarifying the influence of health literacy on SCD risk among older adults would provide valuable information for research into interventions aimed at early dementia prevention. Therefore, in this study, we hypothesized that low health literacy is associated with SCD morbidity in community-dwelling older adults. To test this hypothesis, we explored the associations between SCD and health literacy in a group of community-dwelling older adults.

## 2. Materials and Methods

This study was a cross-sectional survey of community-dwelling older adults administered between September 2016 and September 2017. Participants were recruited in Yasu City, Shiga Prefecture, Japan, in 2016 and 2017 through flyers distributed from June to August. These flyers explicitly stated that participation would be uncompensated. The eligibility criteria were as follows:

1. Older adults aged ≥ 60 years;
2. Not suspected to have MCI or cognitive decline, measured via a Mini-Mental State Examination (MMSE) score ≥ 27 [23].

The exclusion criteria were as follows:

1. Participation in this study for two consecutive years;
2. History of cerebrovascular disease;
3. Past medical history of mental illness;
4. Inability to complete all the questionnaires.

**Figure 1:**
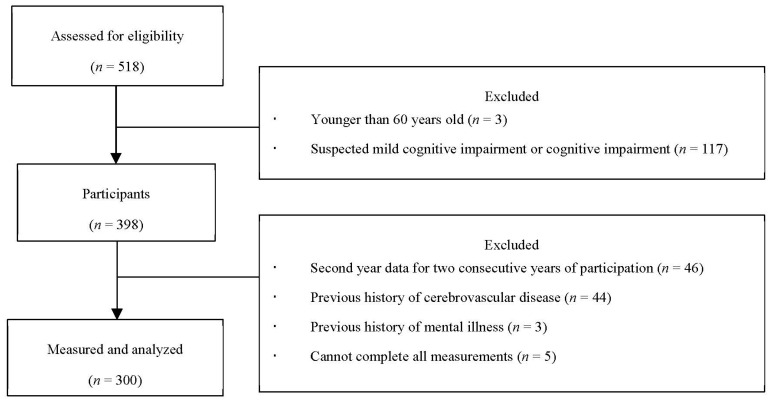
Flowchart of the selection process of the study participants.

Data from 300 participants were ultimately subjected to statistical analysis (Figure 1).

Written informed consent was obtained from each individual prior to participation. This study was conducted in accordance with the Helsinki Declaration and was approved by the Ethics Committee of Kyoto Tachibana University (accession nos. 14–5).

Several other parameters were assessed in addition to the participants’ SCD status. Health literacy was evaluated using the Communicative and Critical Health Literacy (CCHL) scale. Global cognitive function was assessed using the MMSE. Furthermore, depressive symptom severity was evaluated using a five-item version of the Geriatric Depression Scale (GDS-5). Finally, the participants’ basic information (including their sex, age, body mass index [BMI], and educational level) were obtained via an interview. All the measurement tools used in the original Japanese version or translated Japanese versions have been validated and are reliable.

SCD status was ascertained by an interview; the participants were asked three questions that constitute the Cognitive Function domain of the Kihon Checklist (KCL-CF), a self-reported questionnaire meant to assess frailty, which has both established validity [24,25] and reliability [26]. These questions were “Do your family or friends point out your memory loss?”, “Do you make a call by looking up phone numbers?”, and “Do you find yourself not knowing today’s date?” [26]. In previous research, the KCL-CF has been used as a measure of subjective memory complaints [27], subjective cognitive complaints [28], and self-reported cognitive decline [29]. In this study, however, we used the KCL-CF as a measure of the participants’ SCD status. Each positive response (i.e., suggestive of SCD) was awarded one point. The participants who were found to have a total score of greater than one point (KCL-CF ≥ 1) were considered as having SCD.

Their health literacy was evaluated using the CCHL scale [30]. The CCHL consists of three items pertaining to Communicative Health Literacy (1–3) and two that pertain to Critical Health Literacy (4–5), which, respectively, assess respondents’ abilities to: (1) collect health-related information from a diverse range of sources; (2) extract relevant information; (3) understand and communicate the information obtained; (4) judge the credibility of the information; and (5) make decisions based on the obtained information. This screening tool has both established validity and reliability [30]. The participants answered each of the items on a scale ranging from 1 (strongly disagree) to 5 (strongly agree); the average of these five items was then selected for analysis.

Global cognitive function was assessed using the MMSE [31]. The MMSE is a short test that has been extensively used internationally to efficiently assess cognitive functioning. Covering 11 domains, from writing text to copying a drawing, this screening tool is both effective [32] and highly reliable [33]. The participants’ performance was evaluated based on their total MMSE score, which covered all of its measured domains.

Depressive symptoms were evaluated using the GDS-5, a concise version of the GDS [34], which is a self-reported screening questionnaire created with a focus on the characteristic manifestations of depressive symptoms among older adults. This tool has been reported to be valid and reliable [35]. The GDS-5 consists of five questions in a Yes/No format. Each positive response (i.e., those indicating depressive symptoms) was awarded one point, with the sum of all items being used to assess the symptoms’ overall severity.

For the statistical analysis, all demographic and assessment variables were first checked for normality using the Shapiro–Wilk test. Next, these variables were compared on the basis of the participants’ SCD status—that is, SCD (KCL-CF ≥ 1) vs. non-SCD (KCL-CF < 1)—using a χ2 test (sex), Mann–Whitney U test (CCHL, MMSE, GDS-5), or independent-samples *t*-test (all others). Finally, the significance of the association between SCD morbidity and each variable was investigated using a logistic regression model (forced entry). SPSS Statistics software (version 26; IBM, NY, USA) was used for all analyses, with the significance level set to 5%.

## 3. Results

Table 1 shows a breakdown of the study population by their KCL-CF scores, as well as the item-level agreement frequencies. In total, 64.7% of the participants did not demonstrate any symptoms of SCD, while 35.3% provided an affirmative response to at least one item (1 × Yes: 27.3%, 2 × Yes: 8.0%). However, none of the participants answered “Yes” to all three questions. The item with the most frequent positive responses was “Do you find yourself not knowing today’s date?” (25.7%), while the item that received the fewest positive responses was “Do you make a call by looking up phone numbers?” (0.0%).

Table 2 is a summary of the descriptive statistics for each evaluation measure and demographic variable. Data were compared based on the presumed SCD status according to each participant’s KCL-CF score (i.e., SCD vs. non-SCD). Significant differences were observed between these two groups in terms of age, as well as in their CCHL, MMSE, and GDS-5 performances. The groups were not significantly different in terms of their sex, BMI, or educational background.

Table 3 presents the investigation results of the association between SCD status and each indicator using the logistic regression model. Significant associations with SCD morbidity were identified for age (odds ratio [OR] = 1.08; 95% confidence interval [CI] = 1.03–1.14), CCHL scores (OR = 0.52; 95% CI = 0.28–0.96), and GDS-5 scores (OR = 1.49; 95% CI = 1.13–1.96).

## 4. Discussion

In this research, our hypothesis was that low health literacy is associated with SCD morbidity in community-dwelling older adults. The participants with SCD were significantly older than their non-SCD peers; they also had lower health literacy (CCHL), lower global cognitive function (MMSE), and more severe depressive symptoms (GDS-5). Furthermore, old age, low health literacy, and severe depressive symptoms were identified through a logistic regression analysis as independent factors associated with SCD morbidity in community-dwelling older adults.

SCD morbidity (i.e., KCL-CF score ≥ 1) was found in 35.3% of our study population. This rate has been variously estimated in prior research on community-dwelling older adults at 32.5% [28], 34.5% [36], 34.9% [29], 35.4% [37], and 37.6% [38]. Moreover, high response rate to options that suggest SCD was recorded for the following questions: “Do your family or your friends point out your memory loss?” and “Do you find yourself not knowing today’s date?”. However, no participants gave an affirmative (SCD-positive) response to “Do you make a call by looking up phone numbers?”. This tendency is similar to trends reported in previous research on community-dwelling older adult populations [29,36]. Thus, our item-level agreement frequencies for the KCL-CF, along with SCD morbidity, largely agreed with estimates previously reported in the literature.

Statistical comparisons based on SCD status revealed that participants with SCD were older, scored lower on the CCHL and MMSE scores, and higher on the GDS-5 than their unaffected peers (Table 2). Health literacy has been reported to be significantly lower in older adults with MCI than in those without any cognitive decline [12]. Our findings reveal that significantly lower health literacy can be detected even before MCI onset, at the SCD stage, as compared to non-SCD individuals. Significant differences in age [39], cognitive functioning [40,41], and depressive symptoms [42,43] between SCD-positive and SCD-negative adults have been identified in previous studies. Our results support these previous findings, thereby corroborating the demographic and neuropsychological features of older adults with SCD.

The logistic regression analysis revealed that the factors clearly associated with SCD morbidity are CCHL, aging, and GDS-5 (Table 3). Low health literacy has been linked to lack of exercise [44,45], unhealthy eating habits [44], obesity [45], hypertension [46], diabetes [47,48], heart failure [49,50], and metabolic syndrome [51]. Furthermore, these behaviors and conditions are known as risk factors for cognitive decline [52,53]. In addition, individuals with poor health literacy reportedly have low rates of participation in cognitive and social activities, which are known to help maintain cognitive functions [15,16,17]. We speculate that low health literacy in our study population had an influence on the development of SCD, which is a prodromal stage preceding dementia. Our observation of a connection between aging and SCD status is consistent with previous findings that SCD morbidity increases with age [24,54]. The aging process may make individuals more susceptible to SCD as pathologies relating to cognitive decline progress, including amyloid beta deposition [55] and neurofibrillary tangle formation [56], as well as changes in brain functional connectivity [57]. Depressive symptoms have been reported to influence SCD in many previous studies [58,59,60]. Patients with high negative affect (such as when depressed) tend to provide a disproportionate amount of attention to various physical symptoms [61]; therefore, this is one likely reason why depressed patients self-report greater dissatisfaction with certain cognitive skills [62]. However, the MMSE score was not associated with SCD risk according to our logistic regression analysis. A previous study [37] has similarly reported that MMSE scores are not associated with the development of SCD in community-dwelling older adults. This is probably because the MMSE score did not fully capture the early stage of cognitive decline observed in SCD.

This work has a few limitations:

1. Due to this study’s cross-sectional design, we cannot ascertain the causal direction of the relationships discovered. It is also possible that other factors not examined in this study (e.g., low intelligence, low literacy, lack of sophistication, low cognitive reserve, early brain disease) may mediate these relationships. Future longitudinal research with the addition of other relevant factors is thus needed to clarify the relationship between the changes in each of these included measures and SCD onset.
2. Functional aspects were not considered in the health literacy assessment tool used in this study. This topic should be examined using a more multifaceted approach, for example, using instruments that incorporate the functional aspects of health literacy, such as the European Health Literacy Survey Questionnaire [63] or the 14-item Health Literacy Scale for Japanese adults [64].
3. We did not explore any associations with lifestyle habits, lifestyle disease morbidity, or genetic factors. Inactive lifestyle and the prevalence of lifestyle diseases may mediate the association between low HL and the development of SCD. Meanwhile, the presence of genetic factors [65] associated with the development of dementia may influence the development of SCD without being associated with low HL. These topics should be explored in future research.

Owing to these limitations, the generalizability of our findings is limited to within the source population. Despite these limitations, we believe that our findings offer valuable information to healthcare professionals in the field of geriatric medicine and will prove useful in designing effective intervention programs for older adults to prevent dementia.

## 5. Conclusions

In this study, we investigated the relationship between health literacy and SCD status in a population of community-dwelling older adults. Our findings suggest that SCD morbidity is associated with low health literacy, as well as advanced age and depressive symptoms. Healthcare professionals, in the field of geriatric medicine, should carry out targeted measures to address these issues. Some measures that can help address these issues would be improving health literacy and treating depressive symptoms. Despite some limitations, we believe that our findings can assist healthcare professionals in the field of geriatric medicine with the design and implementation of more effective intervention programs aimed at dementia prevention.

## Figures and Tables

**Table 1:** KCL-CF scores and frequency data for SCD items.

Variable	Number of Participants/Frequencies
KCL-CF score (0/1/2/3; *n*)	194/82/24/0
Frequency of subjective memory item endorsement	
Do your family or your friends point out your memory loss?	17.7%
Do you make a call by looking up phone numbers?	0.0%
Do you find yourself not knowing today’s date?	25.7%

KCL-CF: Kihon Checklist—Cognitive Function; SCD: Subjective cognitive decline; *n*: number.

**Table 2:** Comparison of the fundamental information and measurements between the SCD and non-SCD groups.

Variable	Total (*n* = 300)	SCD (*n* = 106)	Non-SCD (*n* = 194)	Effect Size (*r*, *V*)	*p*	
Sex: Male/Female (*n*)	48/252	15/91	33/161	0.04	0.52	a
Age (yr)	73.3 ± 5.8	75.0 ± 5.8	72.4 ± 5.5	0.22	<0.01	
BMI (kg/m^2^)	22.5 ± 3.0	22.6 ± 3.1	22.4 ± 2.9	0.03	0.57	
Educational history (yr)	12.0 ± 2.3	11.7 ± 2.2	12.2 ± 2.4	0.10	0.08	
CCHL (score)	4.0 ± 0.5	3.8 ± 0.4	4.0 ± 0.5	0.19	<0.01	b
MMSE (score)	28.9 ± 1.1	28.7 ± 1.1	29.0 ± 1.1	0.17	<0.01	b
GDS-5 (score)	0.9 ± 1.0	1.2 ± 1.1	0.7 ± 0.8	0.21	<0.01	b

Data are presented as mean ± standard deviation; SCD group: KCL-CF score ≥ 1; non-SCD group: KCL-CF score = 0; *t*-test using independent samples; a: χ2 test; b: Mann–Whitney U test; SCD: subjective cognitive decline; *n*: number; yr: year; BMI: body mass index; kg: kilogram; m: meter; CCHL: communicative and critical health literacy; MMSE: Mini-Mental State Examination; GDS-5: five-item version of the Geriatric Depression Scale.

**Table 3:** Logistic regression analysis with the presence or absence of SCD as the dependent variable.

Variable	B	S.E.	Wald	*p*	Exp (B)	95% CI for Exp (B)	
						Lower	Upper
Sex: Female	1.00						
Sex: Male	−0.32	0.38	0.71	0.40	0.72	0.34	1.54
Age (yr)	0.08	0.02	9.96	<0.01	1.08	1.03	1.14
BMI (kg/m^2^)	0.02	0.04	0.26	0.61	1.02	0.94	1.11
Educational history (yr)	0.00	0.06	0.00	0.98	1.00	0.89	1.13
CCHL (score)	−0.65	0.31	4.42	0.04	0.52	0.28	0.96
MMSE (score)	−0.18	0.12	2.29	0.13	0.84	0.67	1.05
GDS-5 (score)	0.40	0.14	8.16	<0.01	1.49	1.13	1.96

SCD: subjective cognitive decline; B: unstandardized coefficient; SE: standard error; CI: confidence interval; yr: year; BMI: body mass index; kg: kilogram; m: meter; CCHL: communicative and critical health literacy; MMSE: Mini-Mental State Examination; GDS-5: five-item version of the Geriatric Depression Scale.
